# Self-Relevance Does Not Moderate Choice Blindness in Adolescents and Children

**DOI:** 10.1371/journal.pone.0098563

**Published:** 2014-06-02

**Authors:** Melanie Sauerland, Anna Sagana, Henry Otgaar, Nick J. Broers

**Affiliations:** 1 Department of Clinical Psychological Science, Maastricht University, Maastricht, The Netherlands; 2 Department of Statistics, Maastricht University, Maastricht, The Netherlands; University of Tokyo, Japan

## Abstract

In two experiments we tested the choice blindness phenomenon in adolescents aged 11–16 years (Experiment 1, *N* = 87) and children aged 7–10 years (Experiment 2, *N* = 117) for the first time. Analogous to previously reported findings with adult participants, we expected to replicate the robust effect in these age groups. Furthermore, we investigated the hypothesis that self-relevance of choices, defined as the extent to which the self is implicated in a choice, moderates the choice blindness effect in adolescents and children. To this end, we directly compared high and low self-relevance conditions. As expected, the choice blindness effect was robust across age groups. Little support was found for the idea that self-relevance moderates the choice blindness effect. Specifically, no effect of self-relevance on choice blindness was found in adolescents, while the findings in the child sample were inconsistent. Different possible interpretations of the results as well as the possible role of ambiguity for the choice blindness effect are discussed.

## Introduction

Our life is replete with making choices. Each day we have to decide what to wear, what to eat, or more importantly, which doctor to visit. Although most of us would agree that we are aware of those choices and that we can easily detect a mismatch between our choice and the obtained result, recent research indicates quite the opposite. Rather, this research shows that people frequently fail to detect a mismatch between their choice and the achieved outcome when their choice is secretly manipulated. This effect is known as *choice blindness*.

In the first seminal experiment on choice blindness, participants had to decide which of two female faces they found more attractive [1]. Following their decision, participants were handed the selected face and asked to motivate their decision. In three of the 15 trials, however, their choice was manipulated by means of a magical card trick, so that participants ended up with the *non*-selected face. The findings were striking: The overwhelming majority (87%) of the manipulated trials went undetected at the time of the manipulation (i.e., concurrently). After the end of the experiment 70% of the manipulations remained undetected (i.e., retrospectively). Paradoxically, some of the participants included facial features in their motivations that did not correspond to the picture they held in their hands. Conversely, some referred to features that were true only for the manipulated outcome and could not account for the initial choice.

More recent studies investigating the phenomenon picked up on the critique that ratings of facial attractiveness lack personal relevance to the participants [2]. Conversely to the criticism, this research showed that participants can be blind to manipulations of their moral attitudes [3], political attitudes [4] and reports concerning their history of norm-violating behaviors [5]. However, none of these studies provided a direct comparison between more and less self-relevant conditions. Furthermore, the performed choices had no consequences.

A large body of literature on the so-called *self-reference* effect in memory predicts that detection rates should differ as a function of the level of self-relevance of the task. The effect was first demonstrated and defined by Rogers, Kuiper, and Kirker [6] as the tendency of people to encode information in which the self is implicated in a privileged way. The term self-reference has often been used synonymous with personal relevance or self-relevance in the literature [7]–[9] and we will use the latter terms throughout this manuscript. The self-relevance effect has been studied in dozens of research papers and its robustness has been demonstrated across different age groups, stimuli and stimulus presentations as well as encoding and recall conditions [10]. To name just a few examples, memory for self-relevant terms is superior to memory for non-self-relevant terms, as are self-relevant encoding strategies compared to semantic and other relevant encoding strategies [6], [11], [12]. Likewise, flashbulb memories for self-relevant events have been shown to be stronger than for non-self-relevant events [13]; and mock jurors demonstrated better recall and recognition performance for a self-relevant crime (on campus rape), compared to a non-self-relevant crime (prison rape) [8]. The first aim of the current paper was to test whether the choice blindness effect depends on this self-relevance effect. Based on the literature our expectation was that high self-relevance decisions should lead to higher detection rates than low self-relevance decisions.

Our second aim was to extend the choice blindness paradigm to adolescent and child populations. Although the choice blindness effect has been substantiated in a variety of settings (e.g., personal interactions and computer based tasks; in labs, in a supermarket, in a university library) and for a variety of stimuli (visual, auditory, olfactory, gustatory) [1], [14]–[16], it is imperative to assess whether it will also appear in non-adult populations in order to demonstrate and fully understand the robustness of this effect. Drawing from studies that show that children are less likely to detect changes in an environment than adults [17], [18], one might expect detection rates to be smaller in children compared to adults.

This prediction dovetails nicely with developmental work on compliance revealing that young children are more likely to be persuaded by external influences relative to older children and adults [19]. The reason is that memory encoding is less well-developed in young children making them more reliant on others than older children and adults. Based on this work, one might argue that choice blindness manipulations are more likely to have an impact in younger than older children leading to heightened choice blindness effects and smaller detection rates. Indeed, when drawing parallels with developmental work on source monitoring, research clearly suggests that children have more difficulties with monitoring the sources of their memories than adults especially when the sources are similar [20]. In a sense, participants are involved in source monitoring during a choice blindness experiment. That is, participants have to decide whether the manipulated choice is their own choice and recollection or whether it is provided by someone else. Children have more problems with making such memorial decisions rendering them more prone to choice blindness than adults. Furthermore, based on the finding that the self-relevance effect occurs as early as age four to six [21], [22], we predicted that the moderating effect of self-relevance on choice blindness should be present across different age groups, with all age groups displaying less blindness effects in high compared to low self-relevance conditions.

To assess our hypotheses, we tested adolescents (11–16 years; Experiment 1) and children (7–10 years; Experiment 2) and subjected them to two different choice blindness paradigms, using stimuli and procedures that were suited for each age group. Although the variation in stimuli and procedures puts restrictions on comparisons across experiments, these modifications were crucial in order to generate valid high and low self-relevance conditions for the different age samples. Note that, had we used the same task across different age groups, it is likely that these would have differed in terms of how they interpreted the task. To specify the experimental procedures, adolescents (Experiment 1) either indicated their preference regarding five interventions (e.g., new classroom chairs, black boards, vending machines) that were planned to take place in their school environment (high self-relevance), or provided more general estimations (e.g., estimate which of two items was more expensive; low self-relevance). We selected the school environment as a source for our manipulations in this experiment because school life constitutes a fundamental part of adolescents' and children's everyday life. Accordingly, changes in the school environment should generally be of interest to the students. Children (Experiment 2), on the other hand, made five preferential choices regarding two toys (high self-relevance), or made an objective assessment (i.e., which of two animals was faster or which of two foods was sweeter; low self-relevance). This setup was chosen to address children's difficulties in thinking in abstract ways.

Additionally, we explored tendency to act according to social demands as a possible moderator of the choice blindness effect. Indeed, it is possible that blindness to manipulations is associated with construct of social desirability, with people failing to report that they did notice the change in manipulated trials due to their need for approval from the experimenter [23].

### Ethics Statement

The studies reported in this paper were approved by the standing ethical committee of the Faculty of Psychology and Neuroscience (Ethics Committee Psychology, ECP). Written consent was obtained for Experiment 1. In line with the formal requirements as dictated by the ECP for testing minors, oral participant consent was obtained for Experiment 2 and parental written consent for both Experiments 1 and 2.

## Experiment 1

### Method

#### Participants

Participants were eighty-seven German high-school students from one high-school (34 boys; *M_age_* = 14.1 years, *SD_age_* = 1.4, age range: 11–16 years). They provided parental consent forms prior to participation that was on voluntary basis; no monetary awards or course credits were awarded.

#### Self-relevance manipulation

In the high self-relevance condition, participants made choices that referred to changes in their school environment (i.e., new classroom chairs, classroom black boards, etc.). In the low self-relevance condition, the choices referred to the same objects, but no association with participants' high-school was established.

#### Design

A 2 (self-relevance: high vs. low) x 2 (similarity: high vs. low) between-subjects design was employed. Participants were randomly assigned to one of the four experimental groups. The distribution of participants across conditions was 22 (low self-relevance/low similarity), 23 (low/high), 20 (high/low), and 22 (high/high).

#### Independent Variables Concurrent and Retrospective Detection

Across the two experiments reported in this paper, the concurrent and retrospective detection rates served as the dependent variables. Concurrent detection refers to detection immediately after the presentation of the manipulated outcome. In the computer-based Experiment 1, this refers to comments provided on a piece of paper intended for participants to write down the reasons for their selection after each trial. In Experiment 2, where a direct interaction took place between experimenter and child participants, concurrent detection was inferred when children told the experimenter that they had chosen the other toy or that she had made a mistake. Retrospective detection refers to detections that were documented by means of the post-test questionnaire (Experiment 1) or post-test interview (Experiment 2). If, however, participants indicated manipulations that had *not* occurred, no retrospective detection was assumed.

#### Stimulus selection

To ensure that the required choices were self-relevant to high-school students, we conducted a first pilot study, using a survey that consisted of eight questions. These referred to the introduction of a new school logo, classroom wall color, classroom chairs, classroom black boards, school lockers, school plants and school vending machines. Participants indicated how relevant the indicated changes would be to them on a scale ranging from 1 (*not important to me*) to 10 (*very important to me*). Note that we referred to importance here, instead of self-relevance, as this term is easier to understand for high-school students. The following is an example item:

“Your school wants to introduce a day on which all students have to wear the same t-shirt in order to promote student team spirit. How important would the design of this t-shirt be to you?”

Fifty-eight high-school students (12 boys; *M*_age_ = 15.4 years, *SD*_age_ = 0.8, age range: 13–16 years) were recruited through a link to the survey on Facebook. The average rated importance ranged from *M* = 4.74 (school plants) to *M* = 7.24 (classroom chairs). We selected the five interventions that were rated most relevant to the high-school students as stimulus categories. These included classroom chairs (*M* = 7.24, *SD* = 2.47), school t-shirts (*M* = 6.76, *SD* = 2.94), school vending machines (*M* = 6.59, *SD* = 3.08), classroom wall color (*M* = 5.40, *SD* = 2.79), and classroom black boards (*M* = 5.26, *SD* = 2.84). The intervention with the highest ratings (new classroom chairs) was selected as the choice to be made during the manipulated trial. This was to ensure that the manipulated trial constituted an item that was highly self-relevant to the participants.

Two sets of stimuli were created, one with high and one with low similarity stimulus pairs. Specifically, we collected six pictures of different exemplars of each of the five selected stimulus categories (i.e., chairs, black boards, etc.) from search engines on the internet and company websites. We paired all six pictures of one category with each other (i.e., all chairs with other) and presented them to 60 second year psychology students (16 men; 19–51 years, *M*_age_ = 21.7 years, *SD*_age_ = 4.6) who participated in exchange for course credit. Their task was to rate the similarity of each stimulus pair on a scale from 1 (*very different*) to 10 (*very similar*). For each of the five stimulus categories, we selected those pairs that were rated most and least similar for our high and low similarity conditions. The difference between high and low similarity stimulus pairs was significant for all stimulus categories, *t*s(59) ≥7.11, *p*s<.001. The means can be found in Table 1.

**Table 1:** Mean Similarity of the Selected Stimulus Pairs (Experiment 1).

	Similarity			
	Low		High	
	*M*	*SD*	*M*	*SD*
Classroom wall color	3.22	1.84	6.43	2.43
Classroom black boards	3.55	1.66	5.58	2.00
Classroom chairs	4.13	1.88	8.07	1.36
School vending machines	4.38	2.06	6.10	2.05
School t-shirts	4.65	2.01	6.98	1.71

#### Post-test questionnaire

The post-test questionnaire was designed to examine whether participants had noticed our manipulations and refrained from revealing this and was used as a means to establish retrospective detection. First, participants were asked if they had any comments, suggestions or if they had had any problems, and if so what the nature of these problems was. Subsequently, participants were misled to believe that the experiment had employed two conditions: one in which some of their choices had been manipulated and one where this was not the case. Participant then had to indicate which condition they thought they had been assigned to. Those who indicated that they had been in the manipulated condition next specified how many manipulations they had noticed and for which specific picture pairs.

#### Social Desirability Scale-17 (SDS-17)

The SDS-17 [24] contains 17 true/false self-report items that assess an individual's need for approval. The rationale of the instrument is that persons with higher need for approval tend to give more socially desirable responses than the average.

#### Procedure

Participants with parental approval were individually taken out of their class and brought to one of three computers. In the low self-relevance condition, students were told that the experimenters were interested in decision-making and decision outcomes as a cover story. These participants were asked about their preference regarding two objects (i.e., two chairs/black boards/t-shirts etc.). However, no relation with their own school was established. In the high self-relevance condition, students were additionally told that the research group collaborated with the headmaster of their school. Specifically, participants were led to believe that their headmaster was interested in the opinion of the students because he wanted to implement some interventions at their school. Therefore, the students should carefully consider their choices because these would have an effect on the pending decisions. Before proceeding, the experimenters made sure that the participants understood the importance of these choices and their impact on the future.

**Figure 1:**
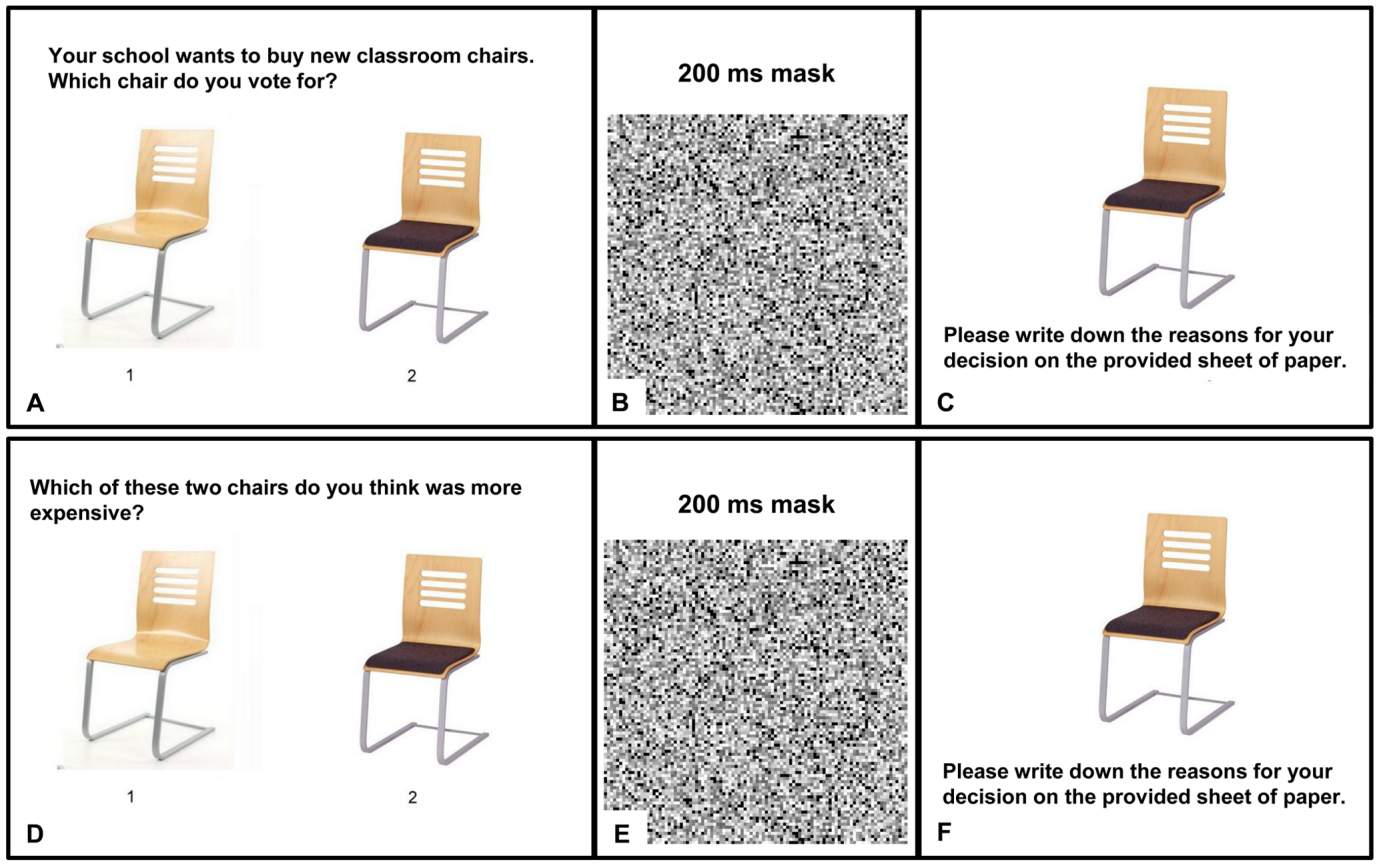
Demonstration of the procedure of a high (A-C) and a low self-relevance (D-F) trial in Experiment 1. **A.** Participants view two stimuli and indicate their choice by selecting 1 or 2. No time limit is imposed. **B.** A masking pattern is presented for 200 ms. **C.** Participants' choice reappears and participants motivate their choice on a separate piece of paper. In a manipulated trial, participants' *non*-choice appears and participants are also asked to indicate motivate their “choice”. **D-F.** Analogous procedure for a low self-relevance trial.

After signing the informed consent form, the computer based experiment [25] started with a practice trial. Participants were shown two objects and had to indicate their preference by pressing the 1 (left object) or 2 key (right object). No time limit was imposed on participants' decision time. Following the decision, a 200 ms mask appeared. Next the selected item reappeared and participants were asked to motivate their choice on a sheet of paper. After the example trial, the five analogous experimental trials followed. In the fourth trial (chairs), however, participants were not presented with their choice, but with the chair they had actually *not* selected. A demonstration of the procedure can be found in Figure 1. After participants were finished with the computer task, they filled in the SDS-17 and the post-test questionnaire. Finally, participants were asked not to talk about the study until it was finished, were offered candy, and thanked for participating. The debriefing took place after termination of data collection.

### Results and Discussion

#### Choice Blindness

The detection rates and 95% confidence intervals for high and low self-relevance conditions across both experiments can be found in Table 2. Concurrent detection in the high self-relevance condition was 11.1%, and 9.5% in the low self-relevance condition. Retrospective detection rates were 57.8% and 54.8%, respectively.

**Table 2:** Concurrent and Retrospective Detection Rates (%) and 95% CIs across Two Experiments.

			Self-relevance			
			High	Low	High	Low
Experiment	*N*	Sample	Proportion concurrent detection (and 95% CI)		Proportion retrospective detection (and 95% CI)	
1	87	Adolescents	11.1 (1.6; 20.7)	9.5 (0.2; 18.8)	57.8 (42.8; 72.8)	54.8 (39.1; 70.0)
2 (Toys)	55	Younger children	50.9 (37.3; 64.5)	50.9 (37.3; 64.5)	54.4 (41.0; 68.1)	52.7 (39.1; 66.3)
	62	Older children	62.9 (50.5; 75.3)	51.6 (38.8; 64.4)	72.6 (61.2; 84.0)	56.5 (43.8; 69.1)
2 (Erasers)	55	Younger children	56.3 (28.9; 83.6)	30.8 (15.6; 45.9)	*not measured*	
	62	Older children	50.0 (32.8; 67.2)	65.4 (45.8; 85.0)		

CI = confidence interval.

To test the effect of age on blindness rates, we split the participants into two age groups (11–13 years vs. 14–16 years). The results showed that neither measure of detection varied as a function of age (concurrent detection: *χ* ^2^(1, *N* = 87)  = 1.95, *p* = .222, *phi* = −.15, retrospective detection: *χ* ^2^(1, *N* = 87)  = 0.49, *p* = .620, *phi* = .07).

Two logistic regression analyses were conducted to establish the effect of self-relevance (high vs. low) and similarity (high vs. low) on concurrent and retrospective detection (yes vs. no). The interactions were non-significant (concurrent detection: Wald χ^2^(1, *N* = 87)  = 2.59, *p* = .108; Nagelkerke's *R^2^* = .08.; retrospective detection: Wald χ^2^(1, *N* = 87)  = 0.98, *p* = .322; Nagelkerke's *R^2^* = .02). Models including only the main effects likewise yielded no significant results (concurrent detection: Wald χ^2^s(1, *N* = 87) ≤0.21, *p*s≥.648; Nagelkerke's *R^2^*s = .01; retrospective detection: Wald χ^2^s(1, *N* = 87) ≤0.52, *p*s≥.472; Nagelkerke's *R^2^*s = .01).

The results for similarity concur with earlier findings [1], [26]. Others, however, reported an effect of similarity for a limited selection of stimuli [14] or for concurrent (but not retrospective) detection [16].

#### Relationship of Choice Blindness and Social Desirability

In accordance with most earlier findings on the relationship between choice blindness and social desirability (Experiments 1 and 2) [5], [16], no significant correlations were obtained for both measures of detection (concurrent detection: *r*(85)  = .12, *p* = .265; retrospective detection: *r*(85)  = .14, *p* = .185). This deems the idea that choice blindness is merely an effect of participants' tendency to comply with the presumed expectations of the experimenter or the tendency to act according to social demands unlikely.

## Experiment 2

A moderating effect of self-relevance on the choice blindness effect was absent in adolescent high-school students (Experiment 1). In Experiment 2, we tested whether self-relevance would moderate the choice blindness effect in children at the elementary school level.

### Method

#### Participants

Participants were *N* = 117 children (51 boys, *M*_age_ = 8.56, *SD*_age_ = 1.07, age range 7–10 years) from three primary schools. Parental consent was obtained prior to participation and testing permission was granted from each school individually. Oral consent was obtained from the child participants.

#### Self-relevance manipulation

Self-relevance was manipulated by varying question type. In high self-relevance trials, children had to indicate a preference. Specifically, they were asked which of two stimuli they liked better (“Which animal [food] do you like better?”). In the low self-relevance condition, the question referred to an objective feature of the animal or food the presented stimulus represented (“Which animal is faster?” or “Which food is sweeter?”).

#### Design

Self-relevance was once manipulated within-subjects (plastic toys) and once between-subjects (erasers). For the (within-subjects) toy manipulation, the order of presentation (high-low self-relevance vs. low-high self-relevance) as well as the stimulus set used for each condition was counterbalanced between-subjects. There was no effect of these two variables on the dependent variables. Table 3 gives an overview of the eight conditions used. For the (between-subjects) eraser manipulation, the stimulus set used (eraser animals vs. eraser foods) was counterbalanced between-subjects next to self-relevance. Preferably, we would have asked child participants which of our two tasks they found more relevant to themselves (indication of preference vs. assessment of fastness/sweetness). However, this did not seem to age appropriate. Alternatively and in line with literature demonstrating that stronger attitudes can be evidenced in faster responses [27], [28] we measured child participants' response times. We expected response times for high-relevance conditions to be faster than for low self-relevance conditions.

**Table 3:** Overview of the Eight Different Conditions and the Procedure in Experiment 2.

Condition	1	2	3	4	5	6	7	8
Eraser trial (a)	Animal*	Animal*	Food*	Food*	Animal	Animal	Food	Food
Plastic toy trial 1^†^	Hoofed*	Wildlife*	Fruit*	Sweet*	Hoofed	Wildlife	Fruit	Sweet
Plastic toy trial 2	Wildlife	Hoofed	Sweet	Fruit	Wildlife*	Hoofed*	Sweet*	Fruit*
Plastic toy trial 3	Fruit*	Sweet*	Hoofed*	Wildlife*	Fruit	Sweet	Hoofed	Wildlife
Plastic toy trial 4^†^	Sweet	Fruit	Wildlife	Hoofed	Sweet*	Fruit*	Wildlife*	Hoofed*
Eraser trial (b): Receive non-selected object from eraser trial^†^								

*Note:* ^†^ =  manipulated trial; * =  high relevance trial.

#### Materials

Three black carton boxes (38.0 cm×19.5 cm×26.5 cm) with an open back and two openings on the top were used to covertly perform the manipulations. The open back allowed the experimenters to see the stimuli; the two openings enabled them to retrieve the stimuli during the experiment. The front side of the boxes (i.e., the side facing the child), displayed the numbers 1 and 2, along with a white line in the middle, indicating two compartments, although there was no actual separator inside the box. Usage of these boxes was practiced with all experimenters prior to testing.

#### Stimulus selection

Two pairs of plastic animals, two pairs of plastic foods toys and a variety of erasers representing animals or foods were used as stimuli. The four plastic toy stimulus pairs were matched in terms of average preference within each stimulus pair, as established in pilot work with 20 child participants (2 boys; *M*_age_ = 8.35, *SD*_age_ = 1.14, age range: 7–10) with written parental consent and oral participant consent. Table 4 presents the mean ranking for each stimulus. The pairs selected for the study were: lion – tiger (wild animals), zebra – horse (hoofed animals), grapes – watermelon (fruits), and chocolate bar – pudding (sweets). According to Wilcoxon signed ranked tests, none of the pairs differed significantly from each other in terms of preference or fastness/sweetness ranking, all |*z*|s≤1.65, *p*s≥.098.

**Table 4:** Mean Ranking Order and Standard Deviation for Plastic Animals and Foods (Experiment 2).

	High self-relevance question		Low self-relevance question	
Stimuli	*M*	*SD*	*M*	*SD*
Lion	3.15	1.39	2.15	1.18
Tiger	2.80	1.28	1.70	0.97
Zebra	2.35	1.22	2.05	0.68
Horse	2.10	1.37	1.70	1.26
Grapes	2.60	1.27	2.60	1.14
Watermelon	2.85	1.84	2.60	1.31
Chocolate Bar	3.45	1.35	3.45	1.35
Pudding	3.60	1.35	3.65	1.26

The pilot participants also ranked two sets of erasers (animals and foods). Based on the results, different combinations of erasers were used for the high and low self-relevance conditions. While we had planned to only use pairs that did not differ significantly in their rankings, this did not always work out when testing in the field. Pairings that significantly differed in their rankings were used in 28 of the cases (23.9%). In order to prevent that a possible effect of self-relevance would be masked by this, we reran the eraser analyses excluding all trials in which an eraser pair had been used that differed significantly in the rankings. The pattern of results remained identical. Therefore, we report the results for the full sample in the results section.

#### Procedure

In order to create a test setting appropriate for children, this experiment did not employ a computer-based task, but included a direct interaction between participant and experimenter. Previous research has shown that choice blindness effects occur in both settings [15].

In their classrooms, participants were told that they could take part in a game in which they would answer questions about different toys. Those children who wanted to participate were then individually led to a different room where two of the six experimenters awaited them. The experiment started with an alleged practice trial containing a pair of foods or animal erasers. Note that the children would receive the eraser they had *not* chosen as a small gift following their participation. This manipulation was conducted to see if children would notice a manipulation after a short delay. After this alleged practice trial, four analogous trials followed. No time limit was imposed on children's decision time.

**Figure 2:**
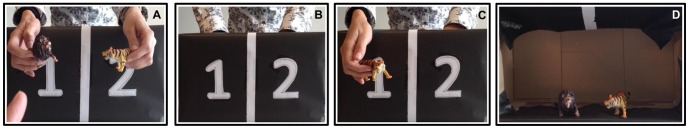
Demonstration of the procedure of a manipulated trial of Experiment 2. **A.** Participants select one of the two presented toys, deciding either which one they like better (high self-relevance) or which of the two animals is faster (low self-relevance). No time limit is imposed. **B.** The experimenter puts both toys back into the box through the two openings on the top and switches the two toys in her hands for this manipulated trial. **C.** The experimenter extracts the non-selected toy from the opening which previously seemed to contain the selected toy. **D.** Rear view of the used box. The absence of a division within the box allows for a switch of the different toys in manipulated trials.

In each trial, the experimenter placed a pair of stimuli into the black box. She then reached through the openings on top and presented the two stimuli to the child. Depending on the condition, the experimenter asked a high or low self-relevance question (i.e., high self-relevance: “Which of the two do you like better?”; low self-relevance: “Which of the two is faster?”; “Which of the two is sweeter?”). After participants made a decision, the experimenter put the two stimuli back into their corresponding openings. Subsequently, she took the selected stimulus out of the box again and asked the child why they had selected that eraser or plastic toy. The occlusion time was as long as it takes to put two stimuli back into a box and take one out again. Although we have not measured the duration, we assume that this part of the procedure took about 1–2 seconds. A trial was concluded by putting the selected stimulus back into the opening and removing both stimuli from the box via the backside. In preparation of the next trial, the experimenter put a new stimulus pair into the box. A demonstration of the procedure can be found in Figure 2. Table 3 illustrates the setup of the eight different administered conditions.

The manipulations were executed in plastic toy trials 1 and 4. In these trials, the experimenter covertly switched the two stimuli inside of the box and retrieved the *non-selected* stimulus from the opening corresponding to the *selected* stimulus. If the child noticed the swap, the experimenter said that she must have made a mistake, retrieved the selected stimulus and asked why they had chosen that object. While one experimenter performed the experiment, a second experimenter measured the children's decision times, wrote down their selections, and documented manipulation detections.

After completion of the plastic toy trials, participants were asked if the experimenter made any mistakes during the experiment and if so during which trial. This post-test interview served as a means of measuring retrospective detection. Finally, participants were thanked for their participation and received the *non*-selected eraser from the eraser trial as a gift. If children at this point said that they had not chosen the eraser they were given as a present, this was counted as concurrent detection. Retrospective detection was not measured for the eraser trials since the manipulation occurred after the post-test interview, from which retrospective detection was inferred. Children were fully debriefed in their classrooms and a debriefing letter was provided for the parents.

### Results and Discussion

#### Manipulation check

As expected, the response times averaged across the two high self-relevance plastic toy decisions were faster (*M* = 2.5 s, *SD* = 1.74) than those for the two low self-relevance plastic toy decisions (*M* = 3.4 s, *SD* = 1.69), *t*(116)  = −5.22, *p*<.001, *d* = −0.52. Additionally, responses to the high self-relevance eraser trial were faster (*M* = 2.3 s, *SD* = 1.94) than to the low self-relevance eraser trial (*M* = 2.9 s, *SD* = 1.84), *t*(115)  = −2.13, *p* = .036, *d* = −0.39. Note that for the analyses of response times, inferential analyses were conducted on log-transformed data (i.e., log base 10) due to significant positive skewness and kurtosis in the response time distribution. The reported means are back-transformed values.

These findings support our idea that judgments about preferences were more self-relevant to our child participants than objective assessments about speed or sweetness. Another possible interpretation of these results could be that the different tasks (semantic memory task vs. preferential task) simply require different amounts of processing time. Future studies could avoid this limitation by using identical tasks for high and low self-relevance conditions while varying self-relevance in terms of incentives (e.g., payment).

#### Choice Blindness and Self-Relevance

The concurrent detection rates in the high self-relevance condition were 57.3% (plastic toys) and 54.7% (erasers), and 51.3% (toys) and 47.9% (erasers) in the low self-relevance condition. Retrospective detection rates for the plastic toys were 54.7% (high self-relevance) and 47.9% (low self-relevance), respectively (Table 2 also includes the means for the two age groups 7–8 years vs. 9–10 years). Recall that retrospective detection was not measured for erasers.

Given that a standard ordinary least square regression analysis would not be a valid statistical model for this design, which includes within-subjects factors and therefore produces correlated data, we opted for Generalized Estimating Equations model (GEE). The GEE model provides an appropriate alternative, as it accounts for the correlated residuals via the specification of a working correlation matrix.

For the plastic toy manipulations, both age (7–8 years vs. 9–10 years) and self-relevance were entered into a GEE analysis as predictors of detection. For concurrent detection, the effect of age was significant, *b* = 1.01, *SE* = .38, Wald χ^2^(1, *N* = 117)  = 6.98, *p* = .008. Specifically, 7–8 year old children were less likely to detect the manipulation (44.5%) than 9–10 year old children (62.9%). The main effect of self-relevance as well as the interaction between both factors were non-significant, Wald χ^2^s(1, *N* = 117)≤2.63, *p*s≥.105. A model containing only the main effects returned similar results.

For retrospective detection, the effect of age was also significant, *b* = 1.15, *SE* = .39, Wald χ^2^(1, *N* = 117)  = 8.80, *p* = .003, with older children displaying higher detection rates (70.2%) than younger ones (48.2%). The effect of self-relevance was non-significant, Wald χ^2^(1, *N* = 117)  = 2.31, *p* = .128. A model containing only the main effects did reveal a significant main effect of self-relevance, *b* = 0.45, *SE* = .22, Wald χ^2^(1, *N* = 117)  = 4.30, *p* = .038. Specifically, high self-relevance manipulations were detected more often (65.0%) than low self-relevance manipulations (54.7%).

For the eraser manipulations (between-subjects factor), a logistic regression analysis was conducted to examine the effect of self-relevance (high vs. low) and age (7–8 years vs. 9–10 years) on concurrent detection (yes vs. no). Both factors as well as the interaction were entered simultaneously. The effect of self-relevance failed to reach significance, but indicated a trend, Wald χ^2^(1, *N* = 117)  = 3.54, *p* = .060, as did the interaction between age and self-relevance, Wald χ^2^(1, *N* = 117)  = 2.94, *p* = .087; Nagelkerke's *R* ^2^ = .08. For pure exploratory reasons we performed post-hoc comparisons which showed that high-relevance manipulations tended to be detected more often (56.3%) than low self-relevance manipulations (30.8%) in younger, χ^2^(1, *N* = 117)  = 3.42, *p* = .081, *phi* = -0.25, but not older children, χ^2^(1, *N* = 117)  = 0.29, *p* = .609, *phi* = 0.07. A model including only the main effects did not reveal any significant effects, *p*s≤.101. One may argue that detection rates for the eraser manipulations might have been deflated due to the fact that children received an eraser as a gift, that is, that children might have kept quiet about a detected manipulation out of fear that then they would not receive a gift at all. If this were the case, however, the detection rates for eraser trials should, on average, be lower than the detection rates for plastic toy manipulations. Inspection of Table 2, however, shows that this was not the case.

## General Discussion

Across two experiments, we sought to extend the existing literature on choice blindness in two ways. More specifically, we aimed to examine the effect of self-relevance on choice blindness rates. Testing non-adult samples for the first time, this research question was implemented across two different age groups, namely in adolescents and children. We expected to replicate earlier findings demonstrating a strong choice blindness effect. Furthermore, we hypothesized that high self-relevance decisions would lead to higher detection rates than low self-relevance decisions across age groups. As such, our two experiments are the first published ones to provide a direct comparison between more and less self-relevant conditions. As predicted, a considerable proportion of participants were blind to our manipulations. Unexpectedly, however, our results do not support the idea that self-relevance can decrease blindness rates [2]. In the following, we will address both of these findings in more detail.

In both experiments we found substantial choice blindness effects, with blindness rates ranging from 37% to 91% concurrently and 27% to 47% in retrospect. The variance across experiments is remarkable, with especially high blindness rates in Experiment 1. One explanation for this might be differences in the immediacy of the consequences following the choices in the high self-relevance conditions. Specifically, in Experiment 2, child participants had to choose between one of two toys, a task that is likely to be of immediate importance at that age. In Experiment 1, however, the consequences of the choices that participants made would only follow in the undefined future. The study of the strength of the choice blindness effect as a function of immediacy of decision consequences might be an interesting alley for future research.

Although we could not make direct comparisons across the two samples, because we had to use different tasks that were suited for both age groups, we were able to make comparisons within children and adolescents. These comparisons showed that children aged 7–8 years old were sometimes less likely to detect our manipulated trials than children aged 9–10 years. This effect only appeared for one (plastic toys), but not the other (eraser) manipulation. Furthermore, we found no effect of age on choice blindness rates within the adolescent sample. One reason for this age trend could be that younger children (aged 7–8) are more likely to be persuaded by external “suggestive” information than older children (9–10-year-olds) [29]. The underlying rationale is two-fold. First of all, younger children's memory is less well-developed than that of older children's. This entails that younger children are more likely to trust other people when talking about their own memories and choices. Relatedly, younger children are more likely to be influenced by authoritarian people (e.g., experimenters) relative to older children [19]. Combined with weaker encoding and source monitoring capacities [20], these mechanisms might have caused the differences in detection rates between the younger and older children that were found for some of our analyses.

Regarding the impact of self-relevance on choice blindness, our data provide only little evidence in support for the idea that self-relevance can decrease blindness rates: In the adolescent sample, no indication for such an effect was found. In the child sample, the findings were inconsistent. Specifically, there was a significant effect in the expected direction for retrospective, but not concurrent detection for the toy manipulation. Furthermore, for the eraser manipulation, we found a marginally significant effect for younger (7–8 years), but not for older children (9–10 years).

Three inferences are possible when looking at the results on the effect of self-relevance on choice blindness: First, one may argue that self-relevance simply does not have an effect on choice blindness. Although we cannot exclude this explanation, it seems unlikely, especially for choices on the high and extreme end of the self-relevance scale (i.e., such choices that one would describe as highly self-relevant). This leads directly to a second explanation of the current results: self-relevance may have an effect on choice blindness, but only for highly self-relevant decisions. Following this argument, the current results would indicate that we did not present our participants with decisions that were self-relevant enough to exhibit the effect. More specifically, it may not be sufficient to only ensure that the high and low self-relevance conditions differ in self-relevance, but self-relevance may also have to reach a high level in order to impact choice blindness rates. This may also explain the tentative support for the self-relevance hypothesis in the child sample. Although we have no objective evidence for this claim, it is possible that the choices made by the children were more relevant to them than those performed by adolescents. Indeed, the experimenters in Experiment 2 reported that the children were quite excited about the task and the choices they had to make. Such excitement was absent in the adolescent sample. Another possibility is that the excitement or interest in the task have an effect on choice blindness.

A third explanation that is worth considering is that another factor interacts with self-relevance. While other factors are conceivable, we would like to focus on ambiguity as such a moderator. Merckelbach, Jelicic, and Pieters [30] argued that evaluative decisions like those made in the choice blindness paradigm hold a certain level of ambiguity, making us prone to choice blindness. Accordingly, we may be unsure about an evaluative decision made at an earlier point [31]. The role of ambiguity in choice blindness has been demonstrated in several studies that showed that blindness occurs not only for choices that are evaluative in nature but also for decisions that rely on long-term episodic memory [5]. Although speculative, it is possible that a self-relevance advantage for detections of secretly manipulated choices only occurs in situations of increased ambiguity. That is, self-relevance may be beneficial when ambiguity is high, but not when ambiguity is low. Following this argument, the current results would indicate that situational ambiguity was too weak in the current experiments for the effect to be revealed. This notion could be tested in future research.

For pure exploratory reasons, we also tested the tendency to act according to social demands as a possible mechanism underlying the choice blindness effect. Consistent with previous studies (Experiments 1 and 2) [5], [16], this idea was not supported by our data. Thus, the current data and the literature to date suggest that social desirability is not a crucial moderator of choice blindness.

To conclude, this work confirms the robustness of the choice blindness phenomenon that is described in the literature across different materials and settings and demonstrates the validity of the effect across the life span. Furthermore, the effect has again shown to be largely unimpaired by different testing conditions. Future research should implement the choice blindness paradigm in settings that are likely to reveal limiting conditions of the effect. Choice blindness remains a fascinating phenomenon which slips in our decisions, both the less, but also the more consequential ones; the one about what to wear, but also which doctor to visit.
